# Mental health outcomes of mothers who conceived using fertility treatment

**DOI:** 10.1186/1742-4755-11-19

**Published:** 2014-02-28

**Authors:** Nikolett Raguz, Sheila W McDonald, Amy Metcalfe, Candace O’Quinn, Suzanne C Tough

**Affiliations:** 1 Department of Obstetrics and Gynecology, University of Calgary, Calgary, Alberta, Canada; 2 Department of Pediatrics, University of Calgary, Calgary, Alberta, Canada; 3 Department of Obstetrics and Gynecology, University of British Columbia, Vancouver, British Columbia, Canada; 4 Department of Community Health Sciences, University of Calgary, Calgary, Alberta, Canada

**Keywords:** Assisted reproductive technologies, In vitro fertilization, Postpartum depression, Anxiety, Fertility treatment

## Abstract

**Objective:**

To compare the proportion of women with self-reported depression and anxiety symptoms at four months postpartum between mothers of singletons who conceived spontaneously and mothers who conceived with the aid of fertility treatment.

**Methods:**

The sample used for this study was drawn from The “All Our Babies Study”, a community-based prospective cohort of 1654 pregnant women who received prenatal care in Calgary, Alberta. This analysis included women utilizing fertility treatment and a randomly selected 1:2 comparison group. The data was collected via three questionnaires, two of which were mailed to the participants during pregnancy and one at four months postpartum. Symptoms of depression and anxiety at four months postpartum were measured using the Edinburg Postnatal Depression Scale and the Spielberger State Anxiety Inventory. Secondary outcomes of parenting morale and perceived stress were also evaluated. Descriptive statistics were used to characterize the population. Chi square tests and in cases of small cell sizes, Fisher Exact Tests were used to assess differences in postpartum mental health symptomatology between groups.

**Results:**

Seventy-six participants (5.9%) conceived using a form of fertility treatment. At four months postpartum, no significant differences were observed in the proportions reporting excessive depression symptoms (2.6% vs. 5.3%, p = 0.50), anxiety (8.1% vs. 16.9%, p = 0.08) or high perceived stress scores (7.9% vs. 13.3%, p = 0.23). Women who conceived with fertility treatment were less likely to score low on parenting morale compared to women who conceived spontaneously and this was particularly evident in primiparous women (12.5% vs. 33.8%, p = 0.01). There were no group differences in proportions reporting low parenting morale in multiparous women.

**Conclusion:**

This study suggests that at four months postpartum, the proportion of women who experience elevated symptoms of depression, anxiety or perceived stress do not differ between mothers who conceive using fertility treatment and those who conceive spontaneously. Parenting morale at four months postpartum is significantly lower in primiparous mothers conceiving spontaneously compared to those who conceive with fertility treatment.

## Introduction

The development of fertility treatments including fertility enhancing drugs and assisted reproductive technology (ART) has made parenthood possible for countless infertile couples. These couples have often undergone a long period of stress and uncertainty before achieving a successful pregnancy and live birth [1]. In women, symptoms of anxiety and depression are common during infertility treatment [2,3].

There is conflicting evidence regarding the psychological state following successful fertility treatment. Some studies have found that symptoms of depression during pregnancy were equivalent among parents of singletons after successful fertility treatment and those conceiving spontaneously [4,5]. Some studies suggest that the anxiety level in the population utilizing ART is decreased compared to controls during pregnancy [4,5]. However, Hjelmstedt et al. demonstrated increased symptoms of anxiety among those who conceived using ART compared to those who conceived spontaneously [6].

Giving birth after a period of infertility may well be the realization of a long awaited ambition but it might not be as unproblematic as it seems. Prior to the successful pregnancy there may have been periods of waiting and frustration. One can extrapolate that these experiences may foster an idealized and unrealistic image of parenthood. After the birth of the child, unmet expectations and beliefs may result in disenchantment and a climate conducive to mental health issues [7,8].

The information about the postpartum mental health of mothers who have undergone fertility treatments is controversial. In their systematic review, Ross et al. [9] indicate that there may be little or no increased risk of postpartum depression in mothers undergoing ART. However, they caution that most of the data on this topic comes from studies with small sample sizes and lack of appropriate comparison groups. They emphasize the need for further research in this area.

This present study aims to evaluate whether the proportion of women who experienced elevated symptoms of depression and anxiety at four months postpartum differed between women who underwent medical interventions to conceive compared to women who conceived spontaneously. Secondary outcomes of parenting morale and perceived stress were also evaluated between the two groups. Finally, using parity as a stratification variable, we investigated whether there were differences in postpartum mental health between primiparous and multiparous women.

## Methods

A total of 1654 women were recruited to take part in the “All Our Babies” (AOB) study in Calgary Alberta Canada. This was a prospective observational cohort study examining use of prenatal and postpartum services, social support and mental and physical health in pregnancy and the postpartum period.

Information on recruitment, data collection and questionnaires utilized in the AOB study is described in detail elsewhere [10,11]. In brief, women were recruited by posters and postcards in the community, at prenatal clinics in Calgary and by Calgary Laboratory Services (CLS) when they went for prenatal blood group serology testing. Women were then telephoned by a CLS representative to enquire if her name and contact information could be released for research purposes. If the woman agreed, she was contacted by a representative of the research team to tell her about the study and to assess her eligibility and her willingness to participate. In order to enrol in the study, participants had to be less than 24 weeks gestation at the time of recruitment, able to answer a written questionnaire in English and receiving prenatal care in Calgary. Three separate questionnaires were sent to participants during the perinatal period. The first questionnaire was mailed to the participants at 18–24 weeks gestation. A second questionnaire was completed by the participants at between 34 and 36 weeks gestations and a third was completed at four months postpartum. Data was collected from May 2008 to August 2010. Overall, the AOB cohort had an 81% retention rate. Of the 1654 participants, we excluded individuals with multiple gestation pregnancies (n = 23), or who did not complete the first questionnaire (included data on method of conception) and the postpartum questionnaire (included data on postpartum mental health outcomes) (n = 335). Our final sample size for analysis was 1296 women.

The first questionnaire collected demographic data, baseline psychosocial and pregnancy data, as well as information on prenatalmental health and experiences of abuse. Information such as age, parity, gravidity, SES, marital status, education, and method of conception was collected. From this data women who utilized medical interventions in order to achieve pregnancy were identified and selected as the study group (n = 76). Medical interventions included fertility drugs (Clomid, Serophine, Gonal-F etc.), artificial insemination (AI), intrauterine insemination (IUI), and assisted reproductive technologies (including in vitro fertilization (IVF), intracytoplasmic sperm injection (ICSI), fresh embryo transfer, donor embryo transfer, superovulation/IUI). The control group (n = 152) was randomly selected from the pool of women enrolled in the study (n = 1220) who did not receive medical interventions to achieve pregnancy. As only a small proportion of the cohort conceived with the help of medical interventions, controls were randomly selected from the AOB cohort on a 2:1 basis using a random number generator.

Pregnancy and postpartum mental health symptoms were measured using standardized scales. Depressive symptoms were assessed using the Edinburgh Postnatal Depression Scale (EPDS). The EPDS is a 10 item self-report scale which was specifically developed as a tool for detecting depression in the perinatal period [12]. It is easily administered and takes about five minutes to complete (Cox et al., [13]). Each item on the scale is given a value between zero and three, with higher scores being consistent with greater depressive symptoms. The total score can range from zero to 30. A total score of 12 or 13 is indicative of a depressive illness [14] (Sensitivity: 84%, Specificity: 75% (Cox et al. [13])). This study utilized a cut-off total score value of 13 or more to identify women with high levels of depressive symptoms, consistent with a clinical diagnosis of major depression [12]. The Spielberger State Anxiety Inventory was employed in this study to evaluate symptoms of anxiety. The inventory contains 20 items which are scored between 1 and 4 with a higher total score indicating greater anxiety (range 20–80) [15]. An established cut-off score of 40 or more was utilized to categorize women as reporting high levels of anxiety symptoms. To assess stress symptoms the 10-item Perceived Stress scale was used. Each item is rated on a five point Likert scales and higher scores indicate greater stress [16]. A cutoff at the 80^th^ percentile was utilized to classify women experiencing high stress symptoms. To evaluate parenting morale, a measure of psychological parental coping resources [17], the Parenting Morale Index was utilized in the postpartum period. This is a ten item questionnaire, with each item being rated on a five point Likert scale. Higher scores reflect better parenting morale [18]. Given no established cut-offs in the literature, we used the 30^th^ percentile of the distribution to classify women as having low levels of parenting morale.

Descriptive statistics were used to characterize the population. Chi square tests and in cases of small cell sizes, Fisher Exact Tests were used to assess differences in postpartum mental health symptomatology between groups. Stratified analysis was used to determine if prenatal and postpartum mental health symptoms differed by parity.

This study was approved by the Conjoint Health Research Ethics Board of the University of Calgary (Ethics ID 20821). Participants provided informed consent at the time of recruitment and were provided copies for their records.

## Results

Of the cohort, 5.9% of women (n = 76) utilized a form of fertility treatment such as ART, fertility enhancing drugs or sperm manipulation techniques to conceive (Table 1). Women who conceived utilizing fertility treatments were significantly older than the population who conceived spontaneously (32.9 vs. 30.8 years of age, p = 0.001) (Table 2). Apart from age, the baseline demographic characteristics of the two groups were similar (Table 2). There were no statistically significant differences between the two groups in terms of previous history of abuse or history of mental disorders (Table 3).

**Table 1:** Type of fertility treatment used (n = 76)

**Type of intervention**	**N (%, 95% CI)**
Fertility-enhancing drugs only	24 (31.6, 20.9–42.3)
Fertility-enhancing drugs and invasive procedure(s) (AI, IUI, IVF, ICSI, embryo transfer, etc.)	27 (35.5, 24.5–46.5)
Invasive procedure(s) (AI, IUI, IVF, ICSI, embryo transfer, etc.) only	25 (32.9, 22.1–43.7)

**Table 2:** Participant demographics

**Variable**	**Fertility treatment group N = 76 n (%)**	**Spontaneous conception group N = 152 n (%)**	**p-value**
Annual household income			0.068
<$60,000	5 (6.9)	23 (15.8)	
≥$60,000	67 (93.1)	123 (84.2)	
Education level			0.428
High school or less	9 (11.8)	13 (8.6)	
Some or completed post-secondary	67 (88.2)	139 (91.4)	
Maternal age at delivery			0.002*
N	74	149	
Mean (Standard deviation)	33.39 (5.11)	31.28 (4.47)	
Time lived in Canada			0.119
Born in Canada/≥5 years	71 (94.7)	134 (88.2)	
<5 years	4 (5.3)	18 (11.8)	
Ethnicity			0.177
Caucasian	55 (72.4)	122 (80.3)	
Non-caucasian	21 (27.6)	30 (19.7)	
Marital status			0.721
Single, separated, divorced, widowed	2 (2.6)	6 (4.0)	
Married, common-law	74 (97.4)	145 (96.0)	
Parity – birth to a fetus > 24 weeks			0.100
No previous births	48 (63.2)	78 (51.7)	
Previous birth to a fetus (At least once)	28 (36.8)	73 (48.3)	
Parity category			0.910
Nulliparous	31 (41.3)	58 (38.4)	
Primaparous	19 (25.3)	41 (27.2)	
Multiparous	25 (33.3)	52 (34.4)	
Delivery method			0.063
Vaginal delivery	52 (68.4)	121 (79.6)	
C-section – planned or emergency	24 (31.6)	31 (20.4)	
Preterm birth			0.345
<37 weeks of gestation	2 (2.7)	10 (6.6)	
37–43 weeks of gestation	72 (97.3)	141 (93.4)	

*Statistically significant.

**Table 3:** Maternal prenatal psychosocial health (measured prior to 24 weeks of gestation)

**Variable**	**Fertility treatment group N = 76 n (%)**	**Spontaneous conception group N = 152 n (%)**	**p-value**
**Depression**			
Depression – categorized by 13 cut-off			0.612
<13	69 (90.8)	140 (92.7)	
≥13	7 (8.2)	11 (7.3)	
**Anxiety**			
Anxiety			0.813
No	63 (84.0)	121 (85.2)	
Yes	12 (16.0)	21 (14.8)	
**Stress**			
Perceived stress score –			0.040*
Low	67 (89.3)	114 (78.1)	
High	8 (10.7)	32 (21.9)	
**Other**			
Ever experienced abuse			0.843
No	54 (72.0)	99 (70.7)	
Yes	21 (28.0)	41 (29.3)	
Previous mental health			0.840
Neither depressed nor mental disorder	53 (69.7)	104 (68.4)	
Either depressed or mental disorder	23 (30.3)	48 (31.6)	

*Statistically significant.

During pregnancy, there were no statistically significant differences in proportions of elevated depression symptoms as scored on the EPDS (Table 3). Nor were there statistically significant differences in the proportions of anxiety symptoms as scored on the Spielberger State Anxiety Inventory between the two groups (Table 3). However, there was a statistically significant difference between the two groups in terms of Perceived Stress. Women who conceived using fertility treatments were significantly more likely to have lower levels of stress during pregnancy than women who conceived spontaneously (10.7% vs. 21.9%, p = 0.04) (Table 3).

Similar to the prenatal period, at four months postpartum, the proportion of women with elevated depression and anxiety scores were not significantly different between the two groups. In contrast to pregnancy, however, statistically significant differences were not observed in the proportion of women with high perceived stress who conceived with fertility interventions and spontaneously (Table 4). Women who conceived with the aid of fertility treatment were less likely to report low parenting morale (19.7 vs. 33.3% p = 0.033) (Table 4). Stratified analysis by parity indicated that the difference in parenting morale between the two groups was only for primiparous women. Among primiparous women, women who conceived with fertility treatment were less likely to report low levels of parenting morale compared to women who conceived spontaneously (12.5% vs. 33.8%, p = 0.01).

**Table 4:** Postpartum psychosocial health

**Variable**	**Fertility treatment group N = 76 n (%)**	**Spontaneous conception group N = 152 n (%)**	**p-value**
**Parenting morale**			
Parenting morale score			0.033*
Low (<37)	15 (19.7)	50 (33.3)	
High (≥37)	61 (80.3)	100 (66.7)	
**Depression**			
Depression – categorized by 13 cut-off			0.502
<13	74 (97.4)	144 (94.7)	
≥13	2 (2.6)	8 (5.3)	
**Anxiety**			
Anxiety at time 3 – categorized			0.075
No	68 (91.9)	123 (83.1)	
Yes	6 (8.1)	25 (16.9)	
**Stress**			
Perceived stress score			0.226
Low	70 (92.1)	130 (86.7)	
High	6 (7.9)	20 (13.3)	

*Statistically significant.

## Discussion

The adverse influence of maternal depression on child health and development has been well documented in the literature, impacting global, social, emotional and cognitive development ([19-22], Lyons-Ruth et al. [23-25]). Infertile women have often undergone a long period of stress before achieving a successful pregnancy and symptoms of depression and anxiety are common during infertility treatment [2,3]. Given the importance of maternal mental health on child health and the commonality of anxiety and depression symptoms during fertility treatment we felt that it was important area of research to examine. In this paperwe examined prenatal and postpartum depression, anxiety, and stress symptoms and postpartum parenting morale between women utilizing fertility treatments to conceive and those conceiving spontaneously. Women who conceived using interventions in our sample had lower levels of antenatal stress and higher levels of parenting morale at four months postpartum than those conceiving spontaneously. There were no differences in elevated depression and anxiety symptoms either prenatally or at four months postpartum.

The rate of excessive depression symptoms, consistent with a diagnosis of major clinical depression in the postpartum period, found in this study is somewhat lower than what has been previously reported in the literature. The range cited in the literature for postpartum depression is about 13–19% [26], whereas in our study the 5.3% of the control population met the criteria for postpartum depression. This difference may reflect the fact in our study the EPDS was administered at the four month postpartum data collection, which may have missed those who would have met the criteria for postpartum depression before or after that time point. Another possible explanation for this difference might be that a proportion of depressed women, due to the nature of the condition, might not have been capable of or willing to participate in the research. Further differences could also be due to methodological differences across studies in terms of assessment tools, cut-off scores, study designs, and sample characteristics.

Anxiety disorders in the perinatal period are not as well studied as depression, yet both impact birth outcomes and child development; however, the prevalence of anxiety disorders during the postpartum period is higher than postpartum depression [27]. The prevalence of high levels of anxiety symptoms in our study population ranges from 8.1% to 16.9% among assisted and spontaneous conceptions respectively. Wenzel et al. [27] found that at eight weeks postpartum, 8.2% of mothers met the DSM-IV criteria for generalized anxiety disorder and 19.7% met most of the DSM-IV criteria. Our rate is in line with other literature. However, our measure reflects excessive levels of anxiety symptoms and not anxiety disorder per se.

In terms of secondary outcomes, the study detected a significant difference in parenting morale, but not perceived stress in the postpartum period between the two groups. Parenting morale was significantly higher in the group that utilized interventions to conceive. Further analysis showed that this was the case only for primiparous women. A possible explanation for this finding might be that the group with the participants conceiving spontaneously for the first time included women who were not planning a pregnancy. In fact, 18% of those conceiving spontaneously reported their pregnancy as being mis-timed in comparison to 5.3% of those using fertility treatments to conceive (p = 0.008). Having an unplanned or suboptimally timed pregnancy may influence preparedness and eagerness to parent, hence influencing enthusiasm for the parenting role, operationalized in this study as parenting morale.

Interestingly, this study found a difference in perceived stress levels between the two groups antenatally but not in the postpartum period. One may speculate that this lower level of antenatal stress in the fertility treatment group may be due to a sense of relief following successfully achieving a long awaited pregnancy. Further research following the trajectory of stress symptoms across time between the two groups, as well as delineation of factors impacting changes in stress would be beneficial.

There are limitations to our study. The data measured in this study were obtained from maternal self-report and this may introduce error. Also, our study population may not be representative of the general pregnant population. The methods we utilized to recruit participants may have selected a highly motivated group, as many of the participants were recruited via posters and postcards. Our questionnaires, although fairly straightforward, were rather lengthy. This might have resulted in a population with a higher education to be more likely to complete the questionnaire. However in comparison with both local and provincial statistics, we have shown that the participants are representative of women giving birth in an urban Canadian centre [10]. Our study was also limited to those participants who could answer a questionnaire in English. This may be a limitation as Sword et al. [28] showed in a Canadian sample that foreign born women are at a higher risk of postpartum depression in comparison to Canadian born women. Furthermore, our study did not examine the effect of mode of conception on rates of high levels of postpartum maternal distress while controlling for other known risk factors for poor mental health in the postpartum period, such as antenatal distress and history of adverse experiences. Another limitation of this study is that different forms of fertility treatment were combined in the analysis and perhaps there is a differential impact on mental health between the different types of treatment. Our sample size was too small for multivariable modelling, therefore further research with larger sample sizes is warranted.

## Conclusion

This study suggests that during pregnancy, there were no statistically significant differences in the proportions of high depression and anxiety symptoms between women conceiving with fertility treatments and those conceiving spontaneously. Women who conceived using fertility treatments were significantly more likely to have lower levels of antepartum stress than women who conceived spontaneously. At four months postpartum, mothers who conceive with fertility treatment do not differ from those who conceived spontaneously in the proportion of elevated depression, anxiety or perceived stress symptoms. First time mothers utilizing fertility treatments to conceive were statistically significantly less likely to report low parenting morale at four months postpartum.

## Competing interests

The authors declare that they have no competing interests.

## Authors’ contributions

SCT is responsible for the overall integrity, progress and timely completion of the AOB study and participated in the design of the study. NLR drafted the manuscript, contributed to the development of the research question, study design and contributed to the interpretation of study results. SWM performed data linkage, and conducted all statistical analyses. NLR, SWM, AM, CO participated in meetings related to study issues and progress as needed. NLR, SWM, AM, CO and SCT were involved in the study design and providing advice on methodological issues. All authors have read and approved the final manuscript.
